# A Magnetic Surfactant Having One Degree of Unsaturation in the Hydrophobic Tail as a Shale Swelling Inhibitor

**DOI:** 10.3390/molecules28041878

**Published:** 2023-02-16

**Authors:** Mobeen Murtaza, Afeez Gbadamosi, Hafiz Mudaser Ahmad, Syed Muhammad Shakil Hussain, Muhammad Shahzad Kamal, Mohamed Mahmoud, Shirish Patil

**Affiliations:** 1 Center for Integrative Petroleum Research, King Fahd University of Petroleum & Minerals, Dhahran 31261, Saudi Arabia; 2 Petroleum Engineering Department, King Fahd University of Petroleum & Minerals, Dhahran 31261, Saudi Arabia; 3 Department of Chemical, Polymer & Composite Materials Engineering, University of Engineering & Technology, New Campus, Lahore 54890, Pakistan

**Keywords:** water-based mud, wellbore instability, shale swelling, shale inhibitor, surfactant, magnetic surfactant

## Abstract

One of the foremost causes of wellbore instability during drilling operations is shale swelling and hydration induced by the interaction of clay with water-based mud (WBM). Recently, the use of surfactants has received great interest for preventing shale swelling, bit-balling problems, and providing lubricity. Herein, a novel synthesized magnetic surfactant was investigated for its performance as a shale swelling inhibitor in drilling mud. The conventional WBM and magnetic surfactant mixed WBM (MS–WBM) were formulated and characterized using Fourier Transform Infrared (FTIR) and Thermogravimetric analyzer (TGA). Subsequently, the performance of 0.4 wt% magnetic surfactant as shale swelling and clay hydration inhibitor in drilling mud was investigated by conducting linear swelling and capillary suction timer (CST) tests. Afterward, the rheological and filtration properties of the MS–WBM were measured and compared to conventional WBM. Lastly, the swelling mechanism was investigated by conducting a scanning electron microscope (SEM), zeta potential measurement, and particle size distribution analysis of bentonite-based drilling mud. Experimental results revealed that the addition of 0.4 wt% magnetic surfactant to WBM caused a significant reduction (~30%) in linear swelling. SEM analysis, contact angle measurements, and XRD analysis confirmed that the presence of magnetic surfactant provides long-term swelling inhibition via hydrophobic interaction with the bentonite particles and intercalation into bentonite clay layers. Furthermore, the inhibition effect showed an increase in fluid loss and a decrease in rheological parameters of bentonite mixed mud. Overall, the use of magnetic surfactant exhibits sterling clay swelling inhibition potential and is hereby proffered for use as a drilling fluid additive.

## 1. Introduction

The most prominent component of drilling field operation is the use of efficient drilling mud for lubricating the drill bit, cuttings transportation from the bottom of the wellbore to the surface, and maintaining the pressure balance in the wellbore being drilled [1,2,3]. Oil-based mud (OBM) and water-based mud (WBM) are the common types of drilling mud [4,5]. Albeit, OBM are more efficient, they are less preferred due to their toxicity, high cost, and dangers they pose to the environment [6]. With the numerous strict regulations currently put in place to combat issues associated with climate change, safe disposal of drilling cuttings from OBM becomes an arduous task [7]. On the other hand, WBM are environmentally benign, have ease of preparation, and cost less [8,9].

A large section of geologic formations drilled for hydrocarbons is characterized by a high percentage of shale layers [10]. The shale layers are composed of clay minerals such as smectite, illite, chlorite, kaolinite, and montmorillonite amongst others which are highly active and reactive [11]. The interaction of the clay minerals with the water component of WBM result in its dispersion to ultra-fine colloidal particles, leading to swelling and hydration behavior [12,13]. This phenomenon reduces the strength of the wellbore and causes other problems such as bit balling, sloughing, pipe sticking, loss circulation, and/or the loss of the entire drilling assembly [14,15]. Hence, shale swelling and clay hydration are widely regarded as the primary cause of wellbore instabilities and can cause a cataclysmic failure such as well blowout when not properly managed, thereby resulting in the significant economic loss [15,16].

To tackle this menace encountered in drilling operations, numerous shale inhibitors have been proffered. Traditionally, inorganic salts such as potassium chloride (KCl), calcium chloride (CaCl_2_), sodium chloride (NaCl), and ammonium chloride (NH_4_Cl) are used for the inhibition of unforgiving shale formations [13,17]. The use of these salts requires a high concentration of the electrolyte to prevent shale swelling and hydration inhibition. However, the use of high electrolyte concentration in drilling fluids leads to the loss of rheological, thixotropic, and cuttings transport properties of the drilling fluid [18]. Moreover, large quantities of electrolytes can cause contaminations and environmental issues. Furthermore, polymers have been examined for their ability to prevent shale swelling and hydration inhibition [18,19,20]. Synthetic and natural polymers were added to the electrolyte as additives to drilling fluids to improve wellbore stability [21,22,23]. Despite the polymer providing the needed viscosity required for improving the rheological properties of the drilling fluid, the macromolecular structure of the polymer form multilayers which cannot intercalate in the clay interlayer and, consequently, limit their efficiency as an additive for shale inhibition [24]. Moreover, the polymers degrade at high temperatures, thereby limiting their properties. The use of particles [25], nanoparticles [26], ionic liquids [27], and polymer nanocomposites have also been explored as additives to drilling fluids for swelling and hydration inhibition with varying degrees of success.

Recently, the use of surfactants has received enormous attention for their shale swelling and hydration inhibition properties. Several classes of surfactants ranging from natural surfactants [28,29], to biosurfactant, anionic, cationic, and Gemini surfactants [30,31,32] have been investigated. Ahmad et al. [33] investigated the use of two cationic Gemini surfactants as shale swelling and clay hydration inhibitors. The two surfactants possess a biphenyl spacer but different counter ions of chloride and bromide groups. They noted that the cationic Gemini surfactant containing chloride and bromide counter ion decreased the linear shale swelling rate by 97.7% and 101%, respectively. Furthermore, the capillary suction time (CST) conspicuously decreased from 1302 s to 312 s and 636 s for the biphenyl chloride and biphenyl bromide Gemini surfactant, respectively. The authors proposed that the hydrophilic ammonium content of the Gemini surfactant attaches via electrostatic force of attraction to the clay content of the wellbore while the hydrophobic part of the surfactant lies on the shale surfactant by forming a protective layer to inhibit hydration.

Lv et al. [34] studied the application of modified biosurfactant for use as a shale swelling inhibition additive via bentonite inhibition test, mud balling test, shale recovery test, and linear shale swelling test. The modified biosurfactant was found to be efficient for the inhibition of highly reactive shale formation. This was achieved by the adsorption of the biosurfactant onto the clay surface by hydrogen bonding and electrostatic interaction, thereby reducing osmotic and surface hydration of clay, and ultimately decreasing water infiltration. Similarly, Ghasemi et al. [35] evaluated the use of henna leaf extract as a natural surfactant for improving the stability of the wellbore by inhibiting shale swelling. The henna leaf extract exhibited better swelling and hydration inhibition behavior compared to inorganic salts by plugging the pore throats of shale. Moslemizadeh et al. [36] modified the surface of montmorillonite clay with cationic cetyltrimethylammonium bromide (CTAB) surfactant and assessed its swelling and hydration inhibition behavior. The CTAB surfactant intercalated into the interlayer space of the clay particles via hydrophobic interaction and cation exchange to prevent the hydration of the clay surface. FTIR confirms the adsorption of the CTAB onto clay, while TGA characterization depicts that the CTAB-modified clay has 4.68% lower water content. Moreover, the experimental result indicates that the affinity of CTAB-modified clay for water decreased by approximately 70%.

More recently, magnetic surfactants, a new class of surfactant, are gaining significant attention due to their magneto-responsive properties [37,38,39]. The surface and interfacial properties of the new surfactant have been perused. As compared to conventional surfactants, the magnetic surfactant is highly efficient in lowering the surface tension and is characterized by low critical micelle concentration [37]. Due to its versatility and good properties, the surfactant has found wide applications in biotechnology, oil remediation, catalysis, water separation, and oilfield applications. Despite its sterling properties, few applications of magnetic surfactant are available due to its recent discovery.

Herein, the in-house prepared magnetic surfactant was applied as a drilling mud additive. The shale swelling and clay hydration inhibition property of this novel surfactant was investigated via linear shale swelling test and capillary suction timer tests. Moreover, the rheological and filtration property of the new class drilling fluid additive was determined. Finally, the inhibition mechanism was investigated by SEM analysis of the filter cake, zeta potential, and particle size distribution analysis. Surfactant interaction with clay was observed by measuring d-spacing and wettability change. For all experiments, bentonite was used as the base mud to assess the properties of the modified mud formulation.

## 2. Methodology

**Figure 1:**
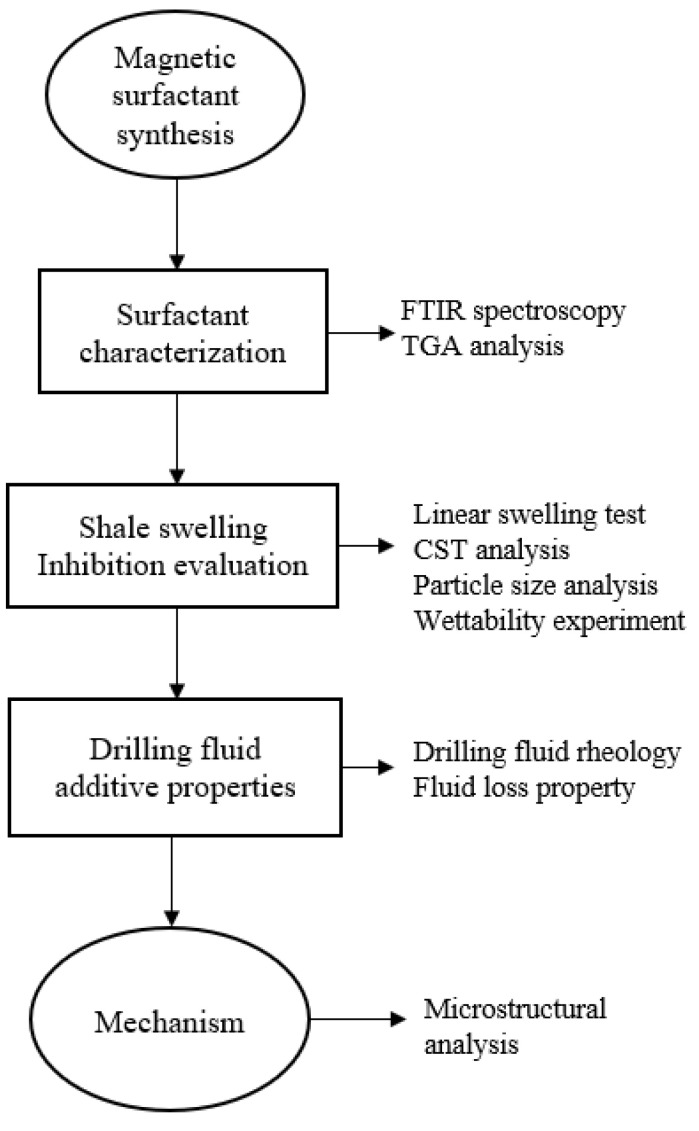
Framework for the evaluation of magnetic surfactant.

Figure 1 presents the framework of this study.

### 2.1. Materials

The materials used in this study are itemized in Table 1. The sodium hydroxide and bentonite were purchased from Sigma Aldrich, St. Louis, MO, USA. The surfactant was in-house synthesized.

### 2.2. Surfactant Synthesis and Characterization

**Figure 2:**
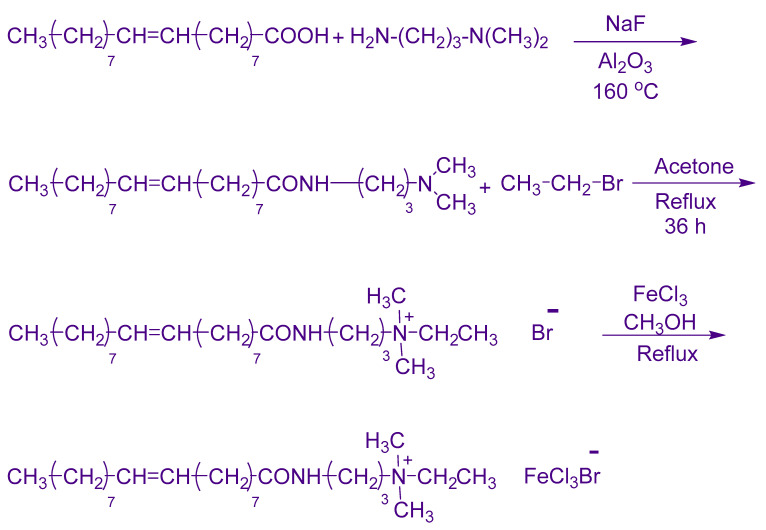
Schematics of magnetic surfactant synthesis.

Magnetic surfactant was synthesized by adopting the procedure mentioned in Figure 2. The FTIR spectra of the in-house synthesized magnetic surfactant were matched with the literature. The detailed synthesis and chemical structure identification of the magnetic surfactant have been given in our preceding report [40].

### 2.3. Solubility Test

In the solubility test, 0.25 wt% of magnetic surfactant was dissolved in distilled deionized water and 0.25 M NaCl solution. The weighted percentage of magnetic surfactant was added to the solution and sonicated for 35 min at 35 °C temperature. Further, the solubility was visually observed over 24 h.

### 2.4. Formulation of Water-Based Mud

To prepare the conventional WBM, 6 wt% of bentonite powder was added to deionized water and constantly stirred with Beach Hamilton mixer, Fann instrument, Houston, TX, USA. Subsequently, the magnetic surfactant-based water-based mud (MS–WBM) was prepared via the addition 0.4 wt% concentration of magnetic surfactant to the base mud. To achieve a good mix, all the mud samples were vigorously stirred for 30 min. Finally, each mud was kept at ambient conditions for 24 h before testing and analysis.

### 2.5. Characterization of the Drilling Mud

The functional group of the formulated drilling mud was determined using Fourier Transform Infrared (FTIR). The transmittance was measured as a function of the wavelength. The transmittance is the fraction of incident light that passes through the sample and comes out on the other side. Moreover, the thermal stability of the mud was investigated via a thermogravimetric analyzer (TGA), PerkinElmer, Waltham, MA, USA. Before the TGA measurement, a purge gas of nitrogen was introduced at the rate of 20 mL/min. Subsequently, the procedure for TGA measurement was executed by placing a small quantity (5 mg) of the dry WBM and MS–WBM on a quartz pan of the TGA and heating the sample from 25 °C to 600 °C at the rate of 10 °C/min.

### 2.6. Shale Inhibition and Swelling Experiment

To assess the shale swelling and clay hydration properties of magnetic surfactant on drilling muds, a linear swelling experiment was conducted using OFITE dynamic swell meter. Before the swelling tests, three shale wafers were prepared using 12 g of bentonite powder in a compactor. The wafers were obtained using the earlier method prescribed in our previous article [30]. The produced wafer was loaded onto the cup assembly of the OFITE swell meter. After that, 120 mL of fluid was gently inserted into the cup assembly and stirred at a shearing speed of 100 rpm for 24 h at ambient conditions. The experiment was repeated for 0.4 wt% of surfactant-based solution. The swelling of each sample was obtained from the in-built software of the OFITE swell meter.

### 2.7. Particle Size Distribution (PSD)

The distribution of additives in the drilling mud is altered due to interaction among each other. The effect of 0.4 wt% magnetic surfactants as shale inhibitors on the dispersion of bentonite particles in deionized water was studied and compared to the conventional drilling mud. Fritsch laser particle sizer ANALYSETTE 22 (Idar-Oberstein, Germany) was used to estimate the particle size distribution of the bentonite material in deionized water. The experiment was repeated by dispersing bentonite in 0.4 wt% magnetic surfactants. Before the particle size distribution measurements, the dispersions were thoroughly mixed with a Beach Hamilton mixer and subsequently left undisturbed for 24 h.

### 2.8. Contact Angle Measurement

The wettability alteration behavior of the conventional and magnetic surfactant-based drilling muds was measured using a Biolin Scientific drop-shape analyzer. To conduct the wettability test, a thin layer of each drilling fluid was coated on a glass plate and allowed to dry for 24 h at ambient conditions. Thereafter, the wettability behavior of the coated clay surface was examined via a small droplet of deionized water. The drop shape analyzer captures the image via a high-resolution camera and measures the contact angle with the aid of in-built software.

### 2.9. Capillary Suction Time (CST) Test

OFITE CST tester was used to evaluate the CST of the drilling fluid. The test is used to determine and quantify the free water in the drilling mud sample. The procedure involves placing 5 mL of conventional WBM and 0.4 wt% MS–WBM in the sample cell. Thereafter, the time it takes to lose free water from the internal electrode to the external electrode in the radial direction was recorded.

### 2.10. Rheological Measurement

To study the flow behavior of the drilling mud against deformation, the rheological measurement of the drilling mud formulations was conducted using a TA rheometer (Newcastle, DE, USA). Before each measurement, the rheometer parts namely the spindle, bob, and cup were thoroughly cleaned with deionized water and blown with air to remove impurities. The viscosity and shear stress of the mud formulation was measured as a function of the shear rate. The shear rate varied from 0.1 to 1000 $s^{-1}$.

### 2.11. Filtration Test

The filtrate loss volume of the formulated drilling fluids was determined using the Fann filter press apparatus OFITE, Houston, TX, USA. The procedure for conducting the filtration test was explained in our previous publication [27]. To conduct the filtration test, Whatman filter paper was inserted into the bottom of the filter cup assembly. Thereafter, 350 mL of the drilling mud was poured into the filter cup and properly fastened. To commence the filtration experiment, compressed air of 100 psi pressure was applied to the filter chamber for 30 min. Consequently, the liquid filtrate was ejected to the bottom of the filtration assembly from where it was collected and estimated. The collected filtrate volume indicates the properties of the drilling mud.

### 2.12. Microstructural Analysis

The conventional drilling mud and surfactant-based drilling mud were analyzed for their structural morphology via scanning electron microscope (SEM). Firstly, the drilling mud samples were dried at 105 °C. Afterward, SEM images were obtained using Helios NanoLab G3 UC manufactured by FEI corporation, Hillsboro, OR, USA. The dried mud sample was titanium coated, placed in the SEM equipment, and scanned using an electron beam. The captured images were subsequently analyzed. X-ray diffraction analysis of the base mud and drilling mud containing 0.4 wt% magnetic surfactant was conducted using a Panalytical Empyrean diffractometer, UK. The dried filter cakes of base mud and 0.4 wt% MS–WBM were used as XRD samples. The mineralogical composition of the samples was performed to examine the d-spacing which was estimated from Bragg’s equation.

## 3. Result and Discussion

### 3.1. Solubility and Salt Tolerance of Magnetic Surfactant

**Figure 3:**
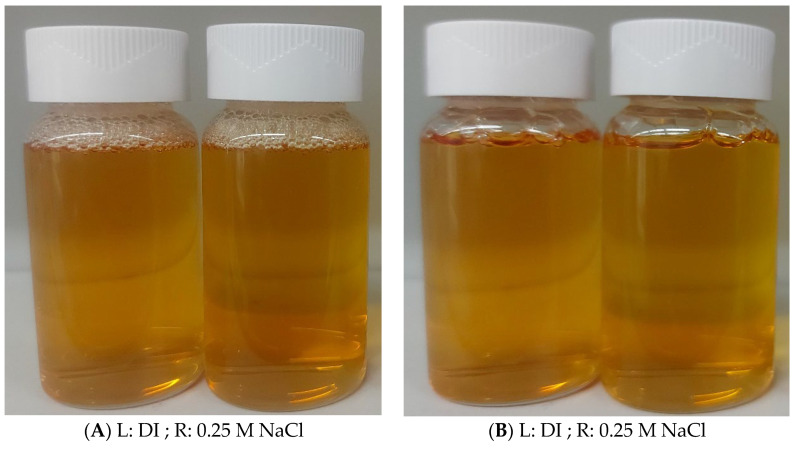
Solubility of Surfactant in DI water and 0.25 M NaCl at the start (**A**) and after 24 h (**B**).

The aqueous solubility and stability of magnetic surfactant against reservoir ions are the fundamental needs for oilfield applications. The in-house prepared magnetic surfactants revealed good solubility in distilled, deionized water as well as in 0.25 M NaCl solution. The surfactant solutions were kept for 24 h. There was no color change observed in the solutions as shown in Figure 3B. It shows that the surfactant was dissolved in 0.25 M NaCl solution and did not precipitate out after 24 h.

### 3.2. Characterization of the Drilling Mud

**Figure 4:**
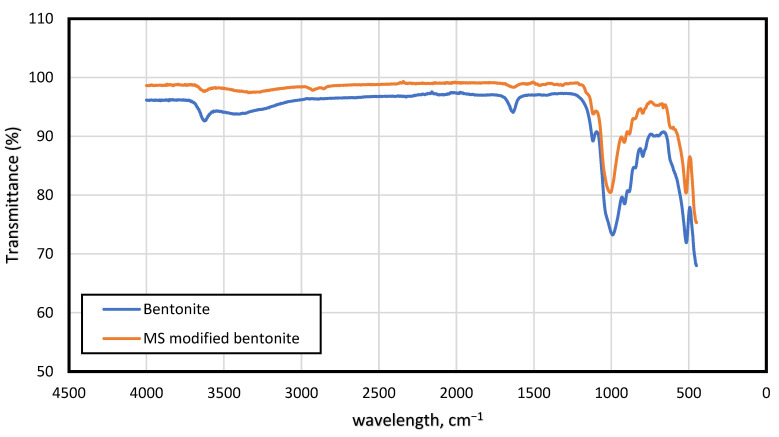
FTIR spectra of bentonite and MS (0.4 wt%) modified bentonite.

FTIR analysis was used to confirm the bonding and structural properties of the drilling mud considered for this study. Figure 4 depicts the FTIR for the base mud (BM) and drilling mud containing 0.4 wt% magnetic surfactant. The wavelength is in the range of 500–4000 $cm^{-1}$ was considered. The reason for conducting FTIR experiments was to investigate the constituent of base mud such as silicates of sodium and the charge attraction of the positive head of magnetic surfactant with negatively charged species in the bentonite clay. According to FTIR results of bentonite clay, the absorption band at 3618 cm^−1^ was assigned to Al-OH-Al in the mineral. The stretching vibration of water (H-O-H) in the bentonite clay was detected at 3422 cm^−1^ and the bending vibration was observed at 1635 cm^−1^. The strong absorption band at 991 cm^−1^ revealed the presence of silicon-oxygen stretching vibration. In the lower region of FTIR spectra, the absorption band 914 cm^−1^ was assigned to the Al-OH stretch, and signals at 881 cm^−1^ were ascribed to the Al-Mg-OH stretching. Moreover, the peak at 515 cm^−1^ was attributed to the Si-O stretching vibration.

The FTIR tests of a mixture of magnetic surfactant (0.4%) and bentonite clay were also conducted and depicted in Figure 4. There is a minor shift of stretching vibration of Al-OH-Al in the bentonite clay from 3618 cm^−1^ (in original form) to the slightly higher region at 3623 cm^−1^ (in the mixture) due to the interactions of bentonite with magnetic surfactant. The two significant peaks at 2922 cm^−1^ and 2851 cm^−1^ indicated the stretching vibration of -CH_2_- and -CH_3_- groups of the alkyl chain of surfactant. Furthermore, the stretching vibration of the C=O group at 1629 cm^−1^ was present, revealing the presence of the carbonyl group in the surfactant molecule. The shifting of silicon-oxygen stretching vibration was also observed from the original position at 991 cm^−1^ to the higher region at 1000 cm^−1^. The shifting of peaks from one region to another was attributed to the interactions of bentonite clay with magnetic surfactant.

**Figure 5:**
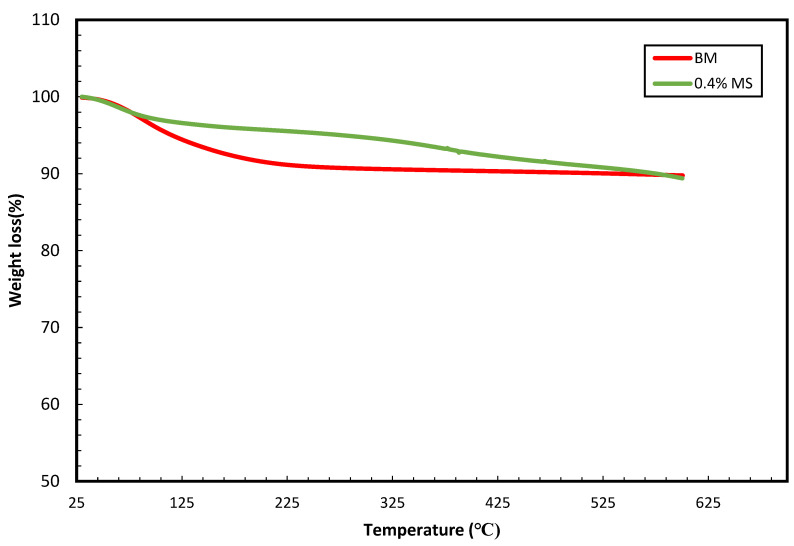
TGA characterization of (i) base mud, (ii) 0.4 wt% MS mud.

TGA experiments are performed to identify weight loss. Weight loss is defined as the breakdown of chemical constituents upon exposure to heat which results in weight loss. TGA analysis was conducted to understand the thermal stability of the conventional WBM and MS–WBM. Figure 5 illustrates the weight loss behavior of the mud over a wide range of temperature conditions (30–600 °C). The conventional BM showed approximately 10% loss at a temperature of around 200 °C due to dehydration of its water content. At the same temperature, 0.4 wt% MS–WBM showed minimal loss with approximately 5%. This indicates better stability of the MS–WBM compared to the conventional WBM. The better thermal stability may be adduced to the intercalation of the hydrophobic content of magnetic surfactant with the sodium bentonite content of the mud which significantly enhanced the hydrophobic properties of the clay, consequently improving its mechanical stability. At a temperature above 300 °C, 0.4% MS–WBM witnessed weight loss due to the thermal degradation of the surfactant chemical structure.

### 3.3. Shale Swelling Inhibition Property

**Figure 6:**
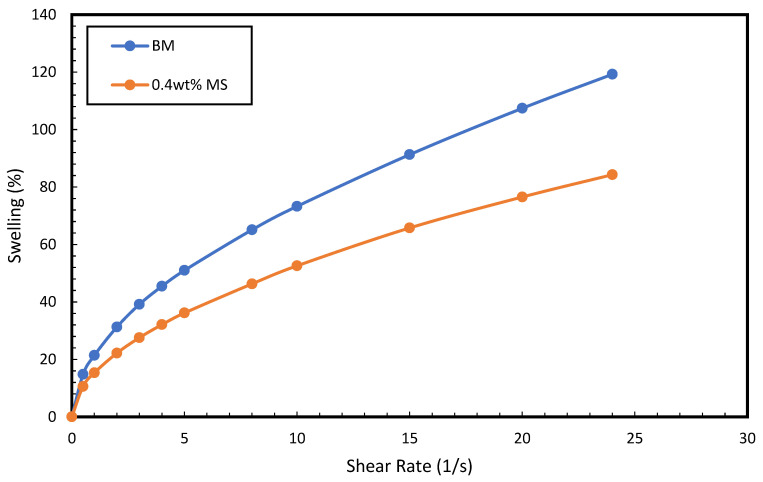
Swelling behavior of sodium bentonite in (i) water, (ii) 0.4 wt% MS.

Swelling is described as the expansion of the shale upon its interaction with water-based drilling mud. The linear swell test of conventional WBM and MS–WBM was investigated over 24 h. The linear swelling rate of the conventional WBM in deionized water is approximately 120%. The addition of surfactant to the base mud was accompanied by a significant reduction in the swelling behavior (Figure 6). Presence of 0.4 wt% MS–WBM reduced the swelling to 84%. This can be adduced to the chemical interaction between the surfactant and the constituents of the drilling mud. Surfactant is usually characterized by a hydrophilic head and hydrophobic tail. The surfactant molecule rearranges itself in the mud such that the hydrophilic head of the surfactant interacts with water molecules present in the drilling mud while the hydrophobic tail forms a layer on the clay surface which minimize the clay swelling behavior of the drilling mud.

**Figure 7:**
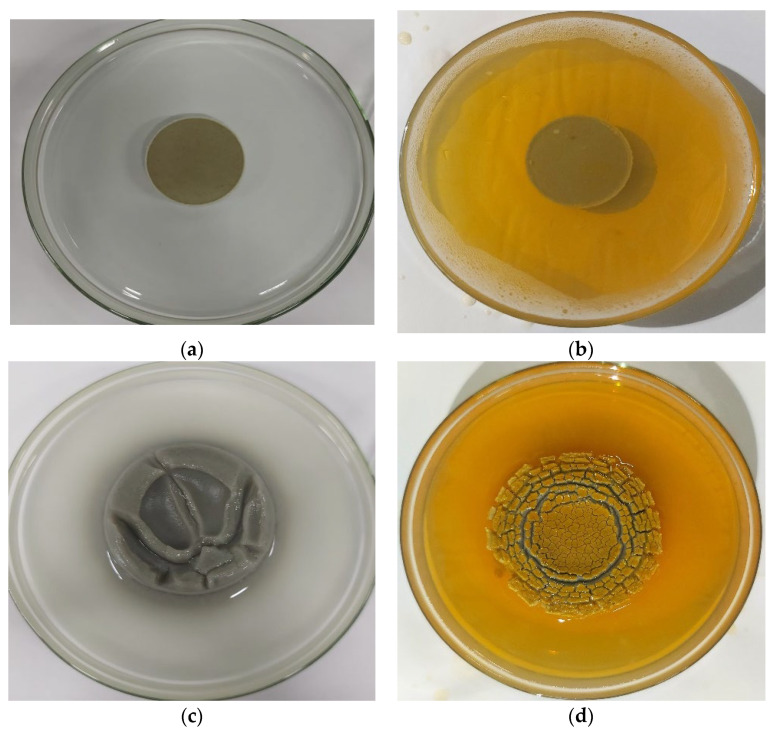
(**a**,**b**) show the bentonite wafers in DI water and MS solution at the start. (**c**,**d**) show the bentonite wafer in DI water and 0.4 wt% MS after 24 h.

Furthermore, free swelling tests were conducted on prepared bentonite wafers by exposing them to deionized water and 0.4 wt% magnetic surfactants on a glass plate and observing the free swelling behavior for 24 h. Figure 7 displays the bentonite wafer before and after exposure to DI water and 0.4 wt% concentration of magnetic surfactants for 24 h. It was observed that the wafers in DI water exemplify the high swelling behavior of the bentonite resulting from the expansion of the particles of the clay. Cation exchange occurs because of attraction between the cations of the clay and water molecules which further pushes the swelling of clay [41]. The expansion resulted in a heightened sample. The exposure of bentonite wafer to MS–WBM exhibits minimal expansion. This is due to the ability of the surfactant to interact with the clay and form a layer that limits the penetration of water into the bentonite particle. The expansion of bentonite in MS solution was radial. The outer layers disintegrated with the core of the wafer remained intact. The result confirms the swelling inhibition behavior of MS molecules in the clay structure.

### 3.4. Particle Size Distribution Analysis

**Figure 8:**
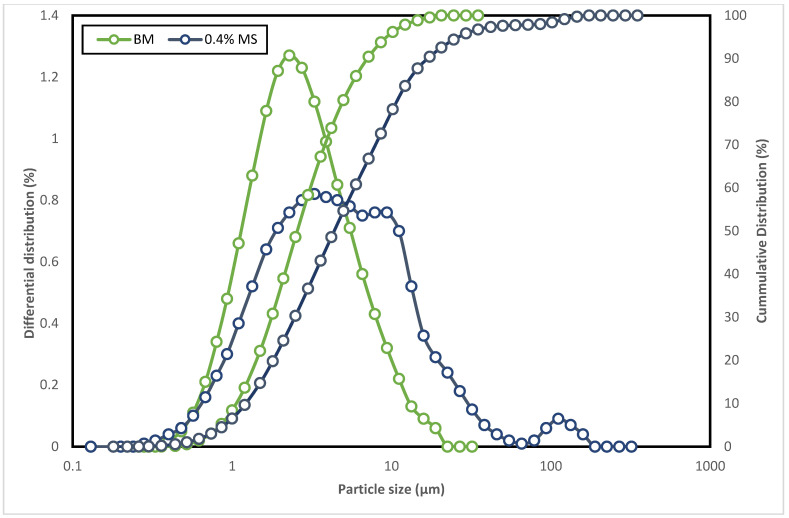
(a) Particle size distribution, and (b) Cumulative density distribution of conventional WBM and MS–WBM.

To further understand the hydration inhibition characteristic of the surfactant on the clay minerals, a particle size distribution analysis was conducted. Figure 8 depicts the particle size distribution and cumulative density distribution of conventional WBM and MS–WBM. Table 2 presents the particle size distribution analysis at different diameters $D_{10}$, $D_{50}$, and $D_{90}$. As compared to MS–WBM, the conventional WBM has a lower particle size and resultantly in a lower cumulative size distribution. This is because, during the interaction of the clay minerals with water, the bentonite swells and delaminates upon hydration which consequently decreases its particle size. Meanwhile, the addition of magnetic surfactant to the drilling mud causes a reduction of the repulsive forces among the particles of the clay. The surfactant molecules expel and substitute the water molecules in the structure of the clay, thus weakening the diffuse double layer. Consequently, the particle size increases due to the closeness among the bentonite particles of the MS–WBM. This implies the addition of the surfactant prevents the dispersion of clay particles and maintains the shale cuttings’ stability [42]. This result corroborates the outcome of the linear shale swelling test.

### 3.5. Wettability Test

**Figure 9:**
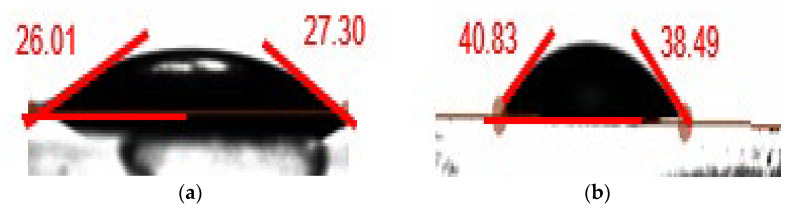
Water contact angle of (**a**) conventional WBM, (**b**) 0.4 wt% MS–WBM.

The wettability alteration measurement of the conventional WBM and surfactant WBM were measured to further probe into the effect of the surfactant on the WBM. As illustrated in Figure 9, the contact angle of WBM on a glass plate is 26.65°. On the other hand, the 0.4% MS–WBM was recorded as 39.66°. The increase in the water contact angle with the addition of surfactant can be adduced to the increase in the hydrophobicity of the clay in the presence of surfactant. This limits the penetration of water molecules for MS–WBM when compared to conventional WBM. This further testifies to the shale swelling inhibition property of the surfactant mixed drilling mud.

### 3.6. Capillary Suction Time (CST) Analysis

**Figure 10:**
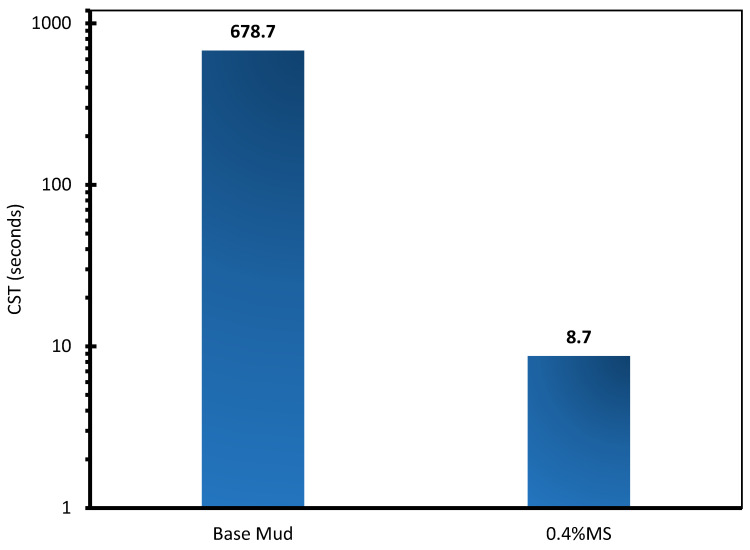
CST analysis of conventional mud and 0.4 wt% MS–WBM.

Figure 10 depicts the CST analysis of the conventional WBM and 0.4 wt% MS–WBM. The conventional WBM recorded a CST of 678.7 s. Meanwhile, the addition of surfactant to the WBM caused a significant reduction in the CST to 8.7 s. Since the presence of surfactant inhibits the penetration of water into the base mud, a shorter time is required for filtrate flow by MS–WBM compared to conventional WBM. The CST data supports the fluid loss results and shows the water-releasing potential of bentonite instead of absorbing and developing high viscosity.

### 3.7. Rheology and Fluid Loss Properties

**Figure 11:**
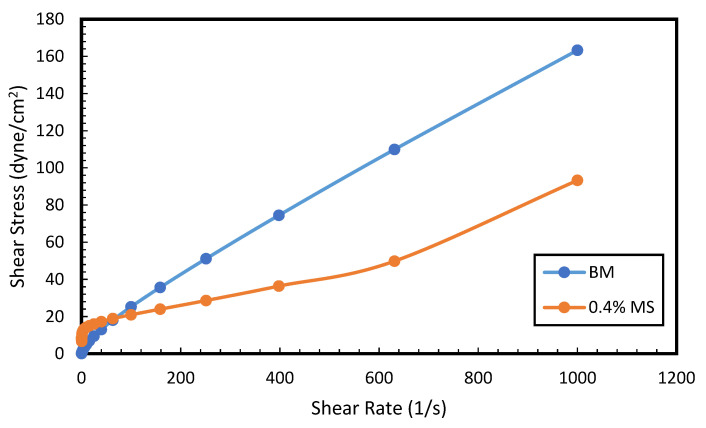
Shear stress versus shear rate (i) base mud, (ii) 0.4 wt% MS. (Temperature = 25 °C).

Figure 11 describes the rheological properties of the conventional WBM and MS–WBM under varying shear rates. Assessment of rheological features of the drilling fluid offers an insight into the flow behavior, hydraulic cleaning, and cuttings transport properties. The drilling mud exhibited pseudoplastic behavior. The shear stress increases in direct proportion to the increase in shear rate. In addition, it was observed that the addition of magnetic surfactant slightly decreases the shear stress of the drilling mud at high shear rates. This may be attributed to the long chains of the surfactant molecule which causes the flocculation of the bentonite particles. Consequently, less swelling of clays was obtained in the presence of the surfactant molecules. It is noteworthy that the decrease in the steady shear is within the allowable limits. Resultantly, low pumping pressure will be required to transport the MS–WBM compared to the conventional WBM. The viscosities and yield points were calculated by applying the Bingham plastic model on the shear rate-shear stress curves. The viscosity of the base mud was 16.60 cP. Upon addition of surfactant, it reduced to 7.56 cP which shows the inhibition of sodium bentonite. The bentonite does not develop viscosity as it could not disperse properly in the water in presence of surfactant. An interesting observation was made in yield points. The yield points were 3.52 dyne/cm^2^ and 10.91 dyne/cm^2^ for WBM and MS–WBM, respectively. The surfactant addition develops a complex attractive structure which results in a high yield point.

**Figure 12:**
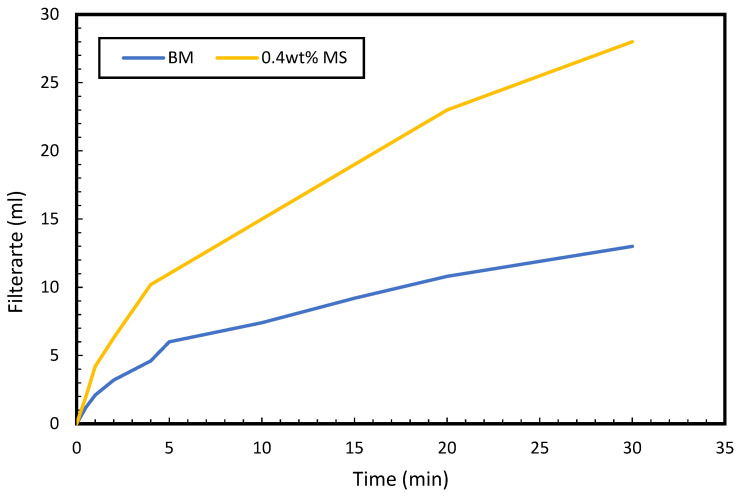
Filtrate volume versus time.

An important property of drilling mud formulation is its filtration property. It indicates the amount of filtrate loss volume from the drilling fluid to the reservoir formation during the drilling operation. Accurate control of filtrate loss can prevent or lessen wall sticking and, in some cases, enhance wellbore stability [43]. The filtration property of the conventional WBM and MS–WBM were measured and shown in Figure 12. The surfactant-based mud displays more filtration volume over time compared to the conventional WBM. At 30 min, the filtrate volume of 28 mL and 12 mL was recorded for 0.4 wt% MS–WBM and conventional WBM, respectively. The higher filtrate volume of the MS–WBM is due to the release of water from the mud. The well-dispersed, bentonite-based drilling mud results in low fluid loss as observed in base mud. Upon addition of surfactant to the base, it inhibits the swelling and dispersion of the bentonite in water. The absorption capacity of the bentonite reduces and water releasing capacity increases upon the addition of surfactant.

### 3.8. Microstructural Analysis

**Figure 13:**
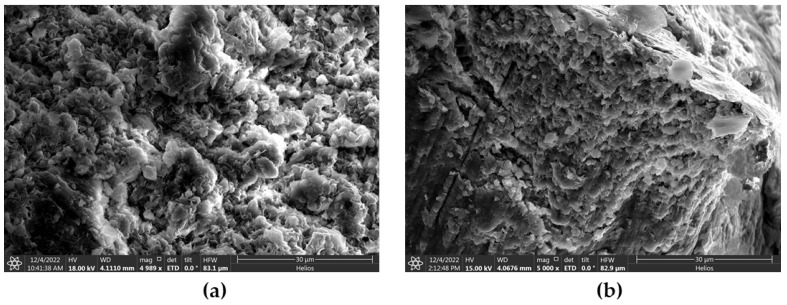
SEM images of (**a**) WBM, (**b**) 0.4 wt% MS–WBM.

To understand the mechanism of hydration inhibition behavior of the magnetic surfactant on the bentonite particles of the drilling mud, morphological and microstructural characterization was carried out for the bentonite and magnetic surfactant-coated bentonite particle via SEM analysis. Figure 13 presents the photographic representation obtained for the WBM and 0.4 wt% MS–WBM under SEM. As depicted in Figure 13a, the conventional bentonite particle of WBM displayed an irregular and flaky structure of the clay particle with easily identifiable small pores and cracks that are easily accessible to water for intrusion [44]. Further, it shows the small, dispersed clay particles. On the other hand, 0.4 wt% WBM exhibited a smooth and orderly structure due to intercalation and adsorption of the magnetic surfactant in the crystalline structure of the clay particle (Figure 13b). As compared to conventional bentonite samples, the magnetic surfactant-coated bentonite displays remarkably lower porosity. This implies lower accessibility for intrusion with a tendency for enhanced wellbore stability [45].

**Figure 14:**
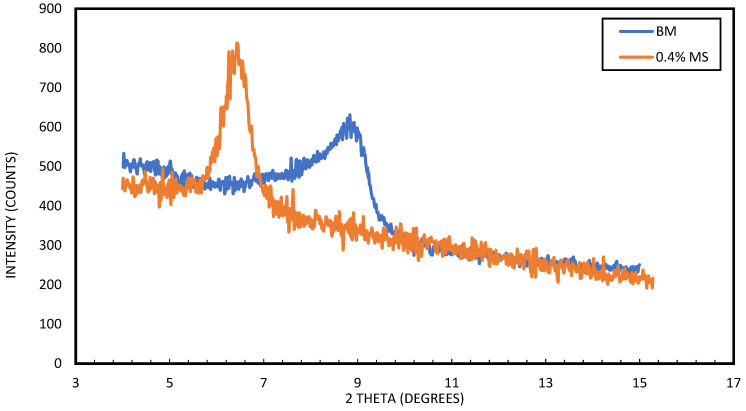
XRD analysis of WBM and 0.4 wt% MS–WBM.

The XRD analysis of the crystallographic structure of base mud and 0.4 wt% MS–WBM is shown in Figure 14. The addition of magnetic surfactant to the base mud caused an increase in the basal d-spacing of the clay interlayer spacing. The basal spacing increased from 12.2 Å to 13.62 Å. This can be attributed to the intercalation of the interstices of the bentonite clay layers which resulted in the inhibition of water molecules and prevented the swelling behavior of the clay.

## 4. Conclusions

The performance evaluation of magnetic surfactant as a shale swelling additive in water-based mud was investigated. Moreover, the rheological and filtration behavior of conventional and MS–WBM was evaluated. FTIR confirms the presence of the functional group of the surfactant in the drilling mud while TGA shows that the MS–WBM has better thermal stability compared to the conventional WBM. SEM images confirm the coating presence of the surfactant on the bentonite particle of the drilling mud with magnetic surfactant. The introduction of surfactant into the water-based mud caused exemplary shale swelling inhibition properties due to the intercalation of the surfactant into the interstices of the bentonite clay particles and forming a hydrophobic film, which reduced the presence of water and enhance the stability of the drilling mud. Moreover, the viscosity reduction and fluid loss increase show the magnetic surfactant’s inhibition potential. Overall, magnetic surfactant showed desirable properties for use as an inhibitor in drilling muds.

## Figures and Tables

**Table 1:** Materials used for formulating the bentonite-based drilling mud.

Additives	Base Drilling Mud	Surfactant Mixed Drilling Mud
Water	350 mL	350 mL
Sodium hydroxide	0.1 g	0.1 g
Bentonite	21 g	21 g
Magnetic Surfactant	-	0.4 wt%

**Table 2:** Distribution of particle size in WBM and MS–WBM.

Formulations	Particle Size Distribution (μm)		
	D_10_	D_50_	D_90_
BM	1.06	2.57	7.09
0.4% MS	1.22	4.39	16.96

## Data Availability

Not applicable.
